# FT-NIR Analysis of Intact Table Grape Berries to Understand Consumer Preference Driving Factors

**DOI:** 10.3390/foods9010098

**Published:** 2020-01-17

**Authors:** Teodora Basile, Antonio Domenico Marsico, Maria Francesca Cardone, Donato Antonacci, Rocco Perniola

**Affiliations:** Consiglio per la ricerca in agricoltura e l’analisi dell’economia agraria-Centro di ricerca Viticoltura ed Enologia (CREA-VE), Via Casamassima 148-70010 Turi (Ba), Italy; adomenico.marsico@crea.gov.it (A.D.M.); mariafrancesca.cardone@crea.gov.it (M.F.C.); donato.antonacci@crea.gov.it (D.A.); rocco.perniola@crea.gov.it (R.P.)

**Keywords:** NIR, PLS, PCA, correlogram, sensory analysis

## Abstract

Fourier-transform near infrared spectroscopy (FT-NIR) is a technique used in the compositional and sensory analysis of foodstuffs. In this work, we have measured the main maturity parameters for grape (sugars and acids) using hundreds of intact berry samples to build models for the prediction of these parameters from berries of two very different varieties: “Victoria” and “Autumn Royal”. Together with the chemical composition in terms of sugar and acidic content, we have carried out a sensory analysis on single berries. Employing the models built for sugars and acids it was possible to learn the sweetness and acidity of each berry before the destructive sensory analysis. The direct correlation of sensory data with FT-NIR spectra is difficult; therefore, spectral data were exported from the spectrometer built-in software and analyzed with R software using a statistical analysis technique (Spearman correlation) which allowed the correlation of berry appreciation data with specific wavelengths that were then related to sugar and acidic content. In this article, we show how it is possible to carry out the analysis of single berries to obtain data on chemical composition parameters and consumer appreciation with a fast, simple, and non-destructive technique with a clear advantage for producers and consumers.

## 1. Introduction

The choice of proper harvest time is especially important in non-climacteric fruits such as grape. The maturity requirements for table grape commercialization rely only on two main parameters: sugars (soluble solid concentration, ° Brix) and acid content (titratable acidity), or their ratio (° Brix/acid ratio) [1,2,3]. Even if the content of sugars and acids is a valuable predictor of quality, there are other factors that strongly influence sensorial judgment [4]. Indeed, the evaluation of table grapes in terms of consumer acceptability is better performed through a sensorial analysis [5]. Among non-destructive techniques, FT-NIR spectroscopy is one of the most advanced concerning instrumentation, applications, accessories, and chemometric software packages [6]. FT-NIR spectroscopy combined with chemometrics has been used as a tool for geographical origin discrimination [7] and quality control [8,9] since this technique allows the development of calibration models that can be used for both the quantitative and qualitative determination of various parameters (e.g., pH, sugars, etc.), including minor components (e.g., polyphenols) [10,11] in various food matrices and beverages. Since FT-NIR spectroscopy can capture variations of both chemical and sensory properties, this technique seems ideal to investigate the influence of sugars and acidic composition on consumer acceptance. As reported in previous studies on intact grape berries, no direct correlation has been found between NIR spectra and sensory analysis [12]. Moreover, the majority of articles involving FT-NIR analysis of grape focuses on berry juice, homogenates, or skin extracts. Scanning of single berries is also possible; however, high coefficients of variation in the FT-NIR spectra are observed when samples are scanned in different positions relative to the FT-NIR source. This variation within the same berry might be linked to dust, different degrees of sun exposure, or simply to variations in the punctual chemical composition in terms of sugar and acidic content. Therefore, a better prediction for a homogenized sample compared to intact berry is usually obtained. To overcome the issues linked to inhomogeneity a larger number of samples must be used to build the NIR prediction models for each variety analyzed. In this work, intact berries of two very different table grape varieties (a white-seeded one and a red seedless one), namely “Victoria” and “Autumn Royal”, have been analyzed using different chemical analytical techniques in conjunction with sensory analysis. Chemometric techniques have been applied to search for a correlation between chemical composition in terms of sugar and acidic content and sensory data. A correlogram graph using Spearman coefficients was built and employed to search for a relationship (in terms of higher correlation) between sensory data and wavenumbers in the NIR spectrum. The attribution of those wavelengths to specific compounds (sugars or acids) was tentatively performed. When a correlation was not found between acceptance and the wavelengths linked to the maturity parameters investigated, we hypothesized that other features such as the texture and appearance of grape, which are known to have an important influence in the assessment of fruit, could have been responsible for the positive acceptance.

## 2. Material and Methods

### 2.1. Chemical Analysis

The table grape berries were collected during the 2018 vintage from the experimental vineyards of CREA Research Centre for Viticulture and Enology of Turi, Southern Italy and were obtained from local companies leading producers of table grape. Two very different table grape varieties: a white-seeded one and a red seedless one, namely “Victoria” and “Autumn Royal”, with an range of maturity levels below and above the minimum required values for grape commercialization (12.5° Brix given a total soluble solid content (TSS)/titratable acidity (TA) over 20:1) [1,2,3] were analyzed. After NIR spectra acquisition, the main parameters for grape quality evaluation were analyzed with the primary methods described in the following lines. Total soluble solid content (TSS, ° Brix) was determined at 20 °C using a digital refractometer Atago PR1 (Atago Co., Tokyo, Japan). Titratable acidity (TA) was measured as tartaric acid (g/L) by titration of grape juice with sodium hydroxide (0.1 N) to an endpoint pH of 7. An HPLC analysis of organic acids was performed on single berry juice using an HP 1100 apparatus (Agilent, Palo Alto, CA, USA) with a diode array detector (DAD) set at 210 nm and a Synergi Hydro-RP 80A column, 250 × 4.6 mm, 4 µm (Phenomenex Inc., Torrance, CA, USA) as stationary phase [13]. A semi-automated method for colorimetric and enzymatic assays was employed for sugar determination on berry juice (SATURNO 150 Crony Instruments). The analysis kit employed was specific for D-fructose and D-glucose. The amount of NADPH formed through the combined action of hexokinase (HK), phosphoglucose isomerase (PGI), and glucose-6-P dehydrogenase (G6PDH), measured at 340 nm, is stoichiometric with the amount of D-glucose and D-fructose in the sample.

### 2.2. NIR Analysis

A Bruker TANGO FT-FT-NIR spectrometer was employed for spectra acquisition and OPUS/QUANT software (Bruker Optik GmbH, Ettlingen Germany) Vers. 2.0 was used for chemometric analysis. In order to build FT-NIR calibration models for the investigated parameters, after the NIR analysis hundreds of samples of fresh table grape for each variety were analyzed with primary methods. Each berry was rinsed in distilled water and gently wiped with paper prior to NIR analysis in order to remove any surface dust and dirt without compromise the superficial wax coating of berries. Each berry was placed on the flat surface of a sample cup with a quartz window and manually rotated in order to record the FT-NIR spectra on three different berry faces. Due to the small dimensions and round shape of the samples, it was not possible to automatically rotate the sample; therefore after each measurement, the berry was manually moved. Since berries were not always able to cover the whole emission source inlet of the instrument, to avoid the record of signals from the air each sample was covered with a hollow metal tube. A background spectrum was automatically recorded prior to each sample while both temperature and humidity were kept constant. After the selection of the most relevant wavelengths to reduce the prediction error associated with spectra noise, different pre-processing techniques were compared in order to eliminate unnecessary physical information and magnify relevant variations in the original spectra. The first derivative (FD) and the vector normalization (VN) were chosen in accordance with their predictive performance for the investigated parameters. With the OPUS/QUANT Bruker software the partial least squares (PLS) regression approach was used for the quantification of changes in the sugar, acid, or sensory-related parameters. After cross-validation, outlier removal, and optimization steps, the final version of the calibration models was obtained with the same Bruker software.

### 2.3. Sensory Characterisation

In order to evaluate preferences among grapes with different degrees of ripeness, a hedonic test was performed [14]. Our panel test was composed of 82 subjects (58 high school students and 24 adults) recruited from subjects already involved in a project with the Council for Agricultural Research and Analysis of Agricultural Economics (CREA) Research Centre for Viticulture and Enology and from the staff of the same research center. Berries for tasting were taken from the central part of each bunch, washed in order to remove dirt and dust, shortly wiped on paper, and kept at 20 °C. The room temperature was set at 20 °C and the tasting was performed in the morning (from 10:00 to 12:00 h). After FT-NIR analysis of single berries, each berry was placed on an identical white plate coded with a two-digit random number and presented in random order. Tasters were asked to evaluate the likeability of grapes with different maturity levels without communicating with each other. Filtered water and water crackers were provided as palate cleansers. Each taster was given a set of each variety of grape samples at different ripeness level, without information about differences, and was asked to rate the samples on a scale of 1 to 10. The significance of the sensory analysis was assessed performing the Kruskal–Wallis non-parametric test followed by the Dunn test as a post hoc test, using a *p*-value < 0.05 [14]. Moreover, in order to highlight the taster’s preference, consumers’ acceptance (%CA) was calculated as follows: number of tasters giving a preference value over 5 divided by the total number of tasters [4].

### 2.4. Structure of Data Sets and Statistical Analysis

In order to search for a relationship between FT-NIR and sensory data, a data set was built. Two FT-NIR data matrices (N_1i_), one for each variety analyzed (*i* = 2), were dimensioned at 350 × 1900. Rows represented the number of samples analyzed (50 berries for each of the seven maturity levels) and columns the parameters for FT-NIR measurements (1899 absorbance values recorded with a 4 cm^−1^ step in the 11,544–3952 cm^−1^ range and one descriptor). N_2i_ represents the corresponding mean values matrices dimensioned at 7 × 1900. Sensory data were collected in two matrices: An S_1i_ matrix was dimensioned at 1722 × 1 in which the rows represented the mean score of preference for each of the maturity levels (seven maturity levels, three berries for each maturity level, 82 judges) and the column was the preference parameter. Moreover, an S_2i_ matrix dimensioned at 7 × 1 (mean values for each of the seven maturity levels × one descriptor) was created. Principal component analyses (PCAs) on the N_1i_ matrix as it was and after the removal of wavelengths with negative correlation with “preference” sensory parameter were performed for the “Victoria” variety. PCAs were performed on the average data matrices (N_2i_) for both “Victoria” and “Autumn Royal” varieties. Moreover, a correlation analysis was performed on the average data matrix obtained by the concatenation of N_2i_ and S_2i_ for each variety. All the statistical procedures described in detail were carried out using the R software environment [15].

## 3. Results and Discussion

### 3.1. Chemical Analysis

**Figure 1:**
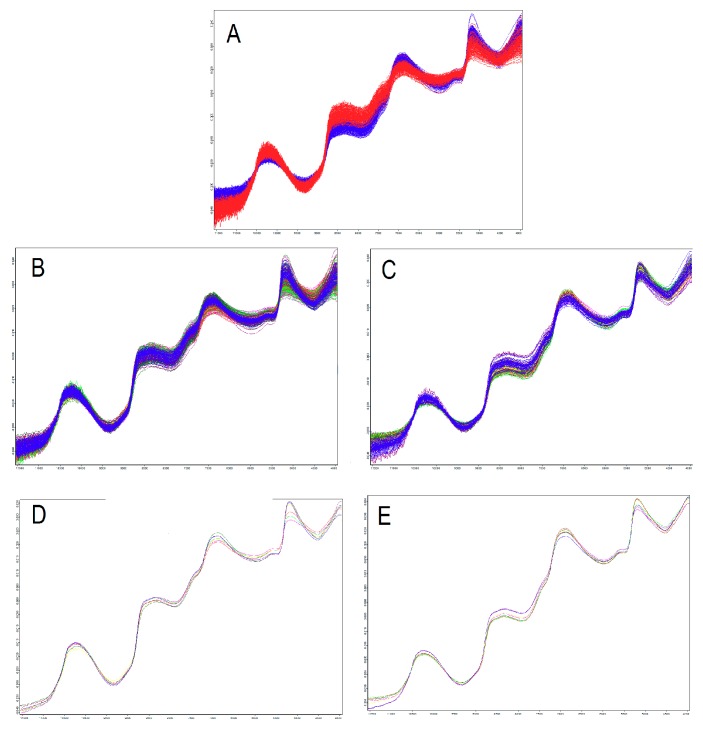
(**A**) Superimposed normalized Fourier-transform near infrared spectroscopy (FT-NIR) spectra (absorbance vs. wave numbers) of single berries for “Autumn Royal” (blue) and “Victoria” (red). Normalized spectra with maturity level-based coloration: “Autumn Royal” (**B**) and “Victoria” (**C**). Averaged spectra for each maturity level: “Autumn Royal” (**D**) and “Victoria” (**E**).

Any direct attribution of specific vibrations to the molecules of sugars was hindered by the nature of the sample. It was not possible to perform an NIR analysis of laboratory*-*prepared samples containing known concentrations of the compound of interest (even in a sample-like medium) since we were measuring intact berries. Moreover, it was not possible to use an internal standard to enhance and thus make more easily identifiable peaks related to a specific overtone vibration to the intact berry. We could have added a sugar (e.g., glucose with a selective deuterium exchange for sugar OH groups) to the juice and then measure the spectrum of the modified sample, but fruit juice spectra and those of intact berry differ. Therefore, as was done by other authors in previous papers, a tentative peak assignment of FT-NIR spectra was done in accordance with the literature. Prominent FT-NIR absorption peaks were observed around 10,200, 8400, 6900, and 5600 cm^−1^ (Figure 1).

Whereas many compounds absorb in the FT-NIR bands highlighted here, those at 10,200 and 6900 cm^−1^ are related to water (second overtone and first overtone of O-H of water, respectively) [16]; this is usually the case for fruits and vegetables and their juices with 70%–80% of water [17]. Sugars and organic acids show absorptions in the same regions of 6978, 5643, and 5614 cm^−1^ [18]. The absorption bands at 6900 cm^−1^ are also related to a combination of stretch and deformation of the O-H group in glucose [19,20]. The 5643 and 5614 cm^−1^ wavelengths are related to the first overtone of CH3 methyl, the first overtone of CH2 methylene, and the first overtone of CH aliphatic, and are attributed to vibrations of the molecules of sugars [21]. C-H overtone and stretch in sugars and organic acids were observed at 8436 and 6978 cm^−1^, whilst the C-O stretch and overtone in sugars and organic acids were attributed to regions at 6978, 5643, and 5614 cm^−1^ [22]. FT-NIR calibration models were built for the investigated chemical parameters. The output of OPUS/QUANT software analysis shows the calibration model together with several statistical parameters such as the root mean square error of cross validation (RMSECV), coefficient of determination (R^2^), and bias. These are a measure for the deviation of the predicted values from the actual values obtained by reference analytical methods. These prediction models could be employed to predict sugar and acid composition of intact berries. The spectra were recorded in the 12,000–3600 cm^−1^ range; a background spectrum was automatically recorded prior to each sample while both temperature and humidity were kept constant. Different pre-processing techniques were compared in order to eliminate unnecessary physical information and magnify relevant variations in the original spectra. The first derivative (FD) together with the vector normalization (VN) was chosen in accordance with their predictive performance. The prediction model obtained with the OPUS/QUANT software (Bruker Optik GmbH, Ettlingen Germany) Vers. 2.0. for TSS shows good predictability (R^2^ = 83%) (Table 1).

**Figure 2:**
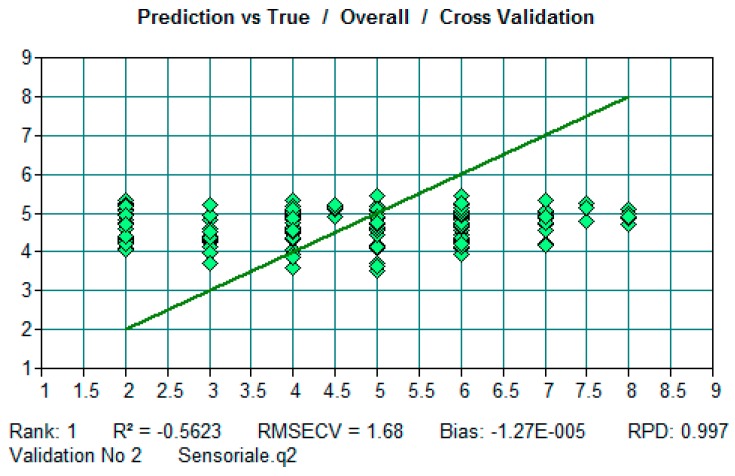
Output of OPUS/QUANT software: Validation curve of “preference” parameter from sensory analysis.

Even if the same number of samples was employed for both TSS and TA FT-NIR analysis, the number of spectra employed to build the model for TA was considerably smaller than those employed for TA analysis. This resulted from the removal of several spectra from the calibration data set which were considered outliers. For TA the predictability of the resulting model was not as good as for TSS. We hypothesize that this lack of prediction ability of FT-NIR can be linked to the gradient of acidity normally present in grape berries (it increases towards the center). Probably, the FT-NIR analysis does not always reach the layer which contains the main acidic compounds; therefore, the acidity value is not accurate enough for the sensitivity of the FT-NIR analysis. We performed the analysis of single organic acids and sugars with HPLC and enzymatic assay. The prediction models built with FT-NIR data did not give satisfactory results for both individual organic acids and sugars. Probably the reason for this lack of predictivity for organic acids is the same as for TA values. Concerning sugars, the number of samples employed was not adequate to determinate a suitable FT-NIR response or the analytical method employed gives a value which is not accurate enough for the sensitivity of the FT-NIR analysis. Under this second hypothesis, a change in the primary method employed for sugar determination would result in a better prediction for single berries. Concerning the “preference” sensory parameter it was not possible to find a good correlation with the PLS regression; therefore, it was not possible to build a suitable model to predict this sensory parameter from NIR spectral features (Figure 2).

### 3.2. Sensory Data Analysis

The analysis of single grape berries with analytical techniques or sensory analysis was performed in conjunction with the record of FT-NIR spectra. This procedure allowed us to have information concerning the chemical composition in terms of sugar and acidic content of each berry (from NIR prediction models) that was employed in the destructive sensory analysis. Sensory data collected from the hedonistic test were analyzed for panel acceptance following the equation: Percentage of consumer acceptance = Number of panelists with a rating > 5/Total number of panelists [4]. From Table 2 it is possible to notice that the consumer acceptance does not decrease linearly with the decrease of ° Brix (nor with the TSS/TA ratio). It is particularly interesting that for the different varieties (“Autumn Royal” and “Victoria” grapes) the acceptance was quite high even with a low sugar content.

Indeed, palatability is a complex result of not only main (sugars and acids) and minor (polyphenols, tannins) components but also other “not flavoring” properties like color and texture related sensations e.g., crunchiness, gumminess, etc. [23]. Among other factors, it is known how the visual appearance of berries is the first quality index for consumers. Indeed, varieties such “Victoria” and “Autumn Royal” with well-defined skin pigmentation even at the beginning of maturation (a grape variety characteristic) show high acceptance values at low glucidic content. Other authors suggest that grape texture is another very important factor in quality judgment and could be used as an indicator for ripening. Indeed, on other fruits such as apples or tomatoes, texture is the main characteristic for determining fruit maturity [23]. In order to understand the importance of sugar and acidic composition and to investigate the presence of other factors influencing the panel judgment on the preference of consumers a deeper statistical analysis was performed (Spearman non*-*parametric test) on sensory and analytical data together (see Paragraph 3.3).

### 3.3. Chemometrics

#### 3.3.1. PCA

**Figure 3:**
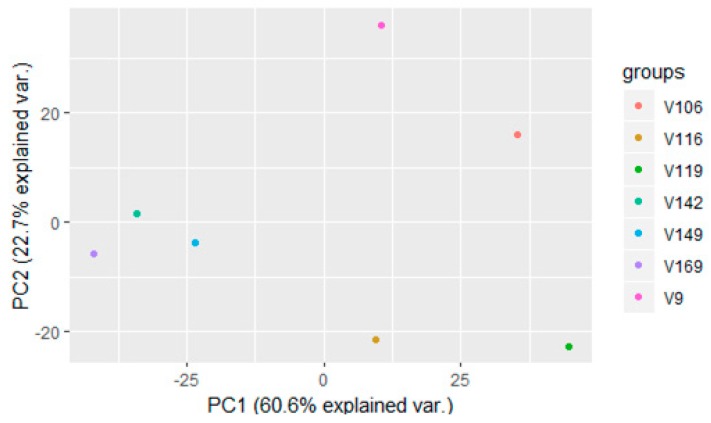
Principal component analysis (PCA) of mean values for the 1900 absorbance values of FT-NIR spectra for the seven maturity levels of “Victoria” grape: 16.9° (V106), 14.9° (V149), 14.2° (V142), 11.9° (V119), 11.6° (V116), 10.6° (V106), and 9° (V9) Brix.

The PCA analysis is a statistical discrimination technique able to highlight common features that allow to group samples with similar composition and thus underlying attributes as shown in previous works [24,25]. Since the PCA performed on the spectra with the spectrometer built-in software (OPUS, Bruker) did not highlight specific features (data not shown), a novel MACRO was created on the OPUS software which allowed for the export of hundreds of spectra files at once in an R readable format. These spectra were processed (truncated and normalized i.e., vector normalization and first derivative) in the same way as those employed in the PLS procedure. From each spectrum, the absorbance values for each of the wave numbers were then inserted in a matrix (*n* × 1900; number of samples × wave number values) and this matrix was employed for further statistical analyses. Since it was not possible to easily differentiate groups of samples with different maturity levels we performed a PCA on averaged spectra for each maturity level. Averaging can reduce noise without compromising data while effectively preserving main features which are responsible for differentiation among samples with different maturity levels. The PCA performed on the mean values of absorbance using the whole FT-NIR spectral window for “Victoria” grape samples with different maturity levels is shown in Figure 3.

**Figure 4:**
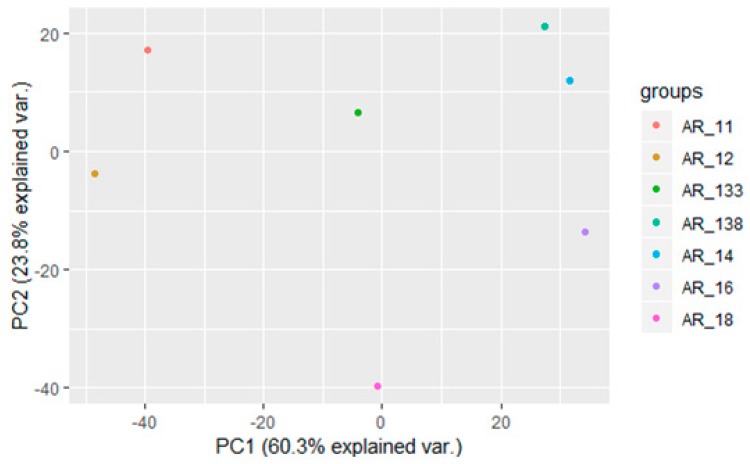
PCA of mean values for the 1900 absorbance values of FT-NIR spectra for the seven maturity levels of “Autumn Royal” grape: 11° (AR11), 12° (AR12), 13.3° (AR133), 13.8° (AR138), 14° (AR14), 16° (AR16), and 18° (AR18) Brix.

Grouping found in the PCA reflects the maturity levels of the samples. Indeed, the lowest maturity samples are grouped in the positive quadrant of the PCA, the intermediate (around 11° Brix) in the second quadrant, the two highest maturity samples are in the negative quadrant, and the 14.2° Brix sample is in the fourth quadrant. Large spectral regions give a strong contribution to the positive PC1 axis: 11,548–10532, 9600–9112, 7208–6488, 5328–4772, and 4228–3952 cm^−1^. Three of these regions can be attributed to water signals; indeed the 10,500 and 6900 cm-1 regions are related to water [16,17], and the 5000–5300 cm^−1^ region is also attributable to water [26]. It looks like that the water content mainly contributes to the positive *x*-axis of the PCA plot. The interpretation of the main contributors of the PC2 axis is quite hard since the loadings are quite low. Among the spectral regions which give a higher contribution to the negative PC2 axis, there is one (4988–4308 cm^−1^) that is likely related to carbohydrates and organic acids [26,27,28,29]. Therefore, we can assume that samples with a negative *y*-axis are characterized by a higher contribution of carbohydrates and organic acids. For the “Autumn Royal” variety a PCA was performed with the mean values of absorbance using the whole FT-NIR spectral window. The PCA (Figure 4) shows that “Autumn Royal” grape samples with 13.8° and 14° Brix are closely related (positive quadrant) as well as the 11° and 12° Brix ones (fourth quadrant), which was expected since the maturity parameters do not differ as much as for the other maturity levels.

The other maturity levels are clearly differentiated, with the 18° Brix sample completely separated from the others, with a highly negative value of the PC2 axis, and one close to zero for the PC1 axis. Among the main wave numbers contributing to the negative PC2 (ranging from −0.5 to −0.9), there are regions at 6772–5324 and 4706–4256 cm^−1^ which can both be attributed to sugar signals [26,30]. Therefore, concerning the PC2 axis, it looks like the influence of sugar content was responsible for the sample’s differentiation. The main FT-NIR regions contributing to the negative PC1 axis (even if with a weight around −0.2) include three of the prominent FT-NIR absorption peaks: 10,200, 8436, and 5614 cm^−1^ (see Figure 1). The absorption at 10,200 is related to water [16], the other two regions are related to sugars and organic acids which show absorptions in the same regions [16,22]. A higher contribution of organic acids and water to the negative PC1 axis explains the grouping of lower maturity samples on the left of the PCA plot.

#### 3.3.2. Correlogram

**Figure 5:**
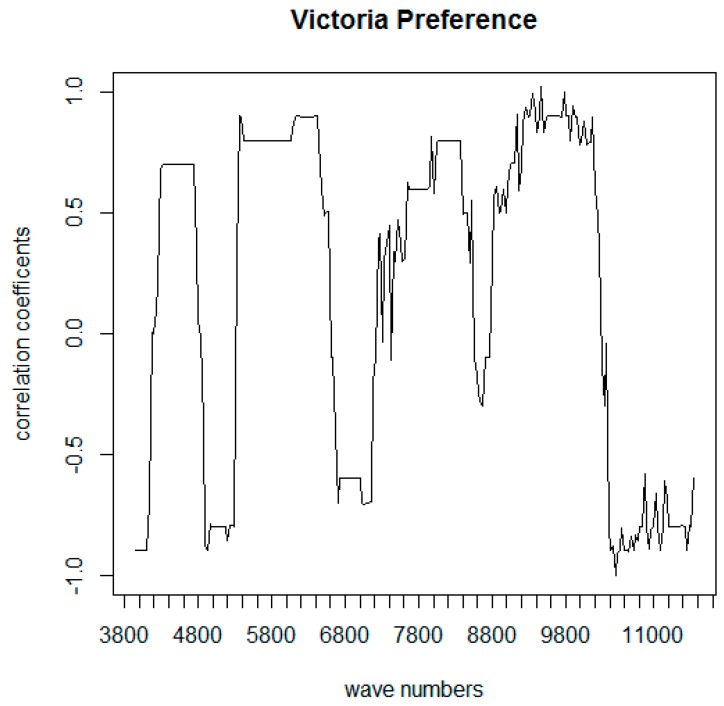
Correlogram: correlation coefficients between FT-NIR spectroscopic data and the sensory data “preference” of “Victoria” grape.

The prediction models built using FT-NIR spectroscopy data in conjunction with sensory analyses did not give satisfactory results, as was previously found in the literature for intact grape samples [12,23]. Even if a direct prediction of sensory attributes from instrumental data was not possible, the exported FT-NIR spectral data were employed to perform deeper statistical analyses. Even a slight difference in grape composition is captured by FT-NIR spectra; therefore, even grape berries of the same variety belonging to the same “maturity level” show different spectral characteristics. To find common features among grape with similar TSS and TA values, which are the only parameters that must satisfy a specific minimum value for grape commercialization, the hundreds of spectra recorded for each maturity level of each variety were averaged. Therefore, the search for a correlation between sensory and instrumental parameters for both “Autumn Royal” and “Victoria” varieties was performed using averaged NIR spectra of samples with the same acidity and sugar content and corresponding acceptance values. To analyze the correlation for each variety a matrix was created binding each N_2i_ matrix (mean values) to a vector containing the mean preference values for each maturity level of the specific variety. To search for a statistically significant relationship (i.e., to measure the existence and the strength of the relationship) between absorbance at each wavelength and acceptance values, the appropriate method is the Spearman rank correlation, since our variables are not normally distributed. To make the outputs more readable, the string containing the Spearman coefficients for sensory descriptor vs. wave-number (the element m_xj_ of the Spearman correlation matrix M_i_ representing the correlation coefficient of the sensory descriptor x and the wavelength j) was plotted in a “correlogram” graph (Figure 5).

**Figure 6:**
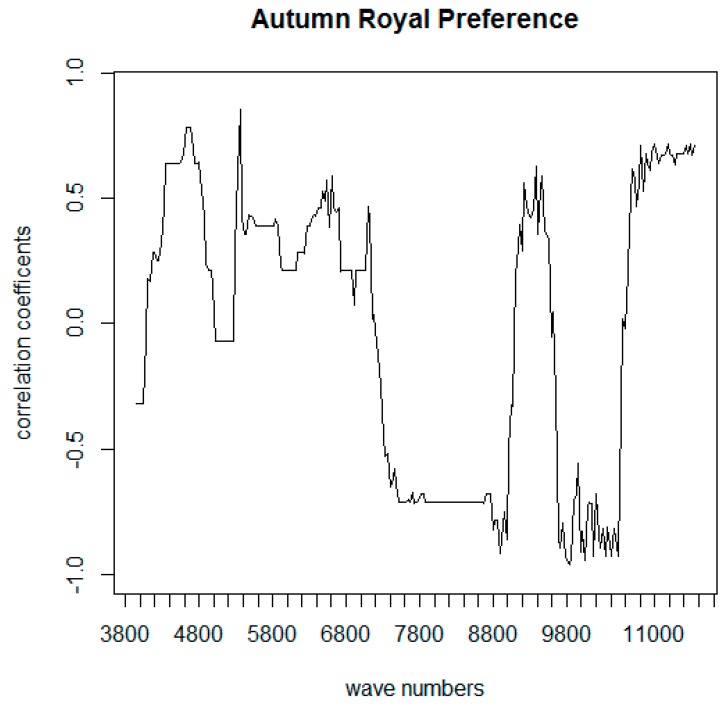
Correlogram: correlation coefficients between FT-NIR spectroscopic data and the sensory data “preference” of “Autumn Royal” grape.

The highest correlation in the graph of the correlation coefficients between preference and each wavelength of the FT-NIR spectra in Figure 5 was associated with wave numbers in the following regions of the FT-NIR spectra: 4000–4500, 5500–6500, and 9000–10,000 cm^−1^, which are all likely related to overtones of carbohydrates and organic acids [16,17,26,28,29]. Therefore, it seems that the sugar and acidic composition of tested grapes directly influenced the preference of the panel. The graph of the correlation coefficients between preference and each wavelength of the FT-NIR spectra for “Autumn Royal” (Figure 6) showed the highest correlation coefficient (*R* = −0.97) for 5372 cm^−1^; this is likely linked to sugars since spectral regions between 5800 and 5400 cm^−1^ are related to the first overtone of C-H stretching and C-H + C-H and C-H + C-C combination bands, respectively, both attributed to vibrations of the molecules of sugars [26,27,28,29,31].

Moreover, the region from 4600 to 4000 cm^−1^ can be ascribed to combinations of O-H bend/hydrogen-bonded O-H stretch (4428 cm^−1^), O-H stretch/C-C stretch (4393 cm^−1^), and combinations of C-H/C-C (4385–4063 cm^−1^) vibrations of the sugar molecules [21]. Besides the positive correlation for the low wave numbers regions, the rest of the correlogram of “Autumn Royal” differs strongly from the “Victoria” one. Strong peaks between 7400 and 6400 cm^−1^ are mainly related to the first overtone of O−H stretching bands of water and while for “Victoria” they are highly negatively correlated (−0.7), for “Autumn Royal” just a slightly low positive correlation (+0.25) is observed. The other regions showing different correlations are: 8500–7500 cm^−1^ positive for “Victoria” and negative for “Autumn Royal”, 9610–10,300 cm^−1^ positive for “Victoria” and negative for “Autumn Royal”, and 10,490–11,548 cm^−1^ negative for “Victoria” and positive for “Autumn Royal”. It seems clear that the preference for “Victoria” and “Autumn Royal” is not correlated to the same spectral signals and thus it was not influenced by the same grape features. This difference is not surprising because these two cultivars are very different in terms of overall appearance (one is red and seedless while the other is white seeded) and composition.

## 4. Conclusions

The objective of this research was the analysis of intact table grape berries to search for a relationship between the chemical composition in terms of sugar and acidic content and the consumers’ preference using near-infrared spectroscopy. The analysis of single berries with analytical techniques or sensory analysis was performed in conjunction with the record of FT-NIR spectra; this allowed to us obtain information on the chemical composition in terms of sugar and acidic content of each berry and, at the same time, to link it to the sensory appreciation of the berry. To search for a correlation between consumers’ preference parameter and chemical composition in terms of sugar and acidic content a correlogram graph was built for each of the two different grape varieties analyzed: “Victoria” (white-seeded) and “Autumn Royal” (red seedless). Vibrational transitions of different chemical compounds in the sample are responsible for absorbance peaks at different wavenumbers of a NIR spectrum. Since in the correlograms of the two varieties the spectral regions with a higher correlation with preference were different, it seems that the reason behind consumers’ appreciation of these two different varieties can be related to different grape characteristics. The attribution of NIR spectra to specific compounds is not simple due to overlapping signals of different groups and the presence of strong absorbing molecules (e.g., water) in fruit samples. A PCA analysis performed for each variety using the absorbance values of the FT-NIR spectra as factors showed how it was possible to group grape of the same variety based on different values of TSS and TA. We searched for a possible explanation of the grouping observed in the PCA plot based on the wave-number regions with higher loadings on the PCA plot axes which have been linked to water, sugar, and acidic composition. Therefore, the PCA analysis was then employed as a tool to link wavelength regions to compounds responsible for the signals in the NIR spectra. The wavelength regions and chemical compounds responsible for those signals were used to interpret the correlograms. It was observed that while for the correlogram of “Victoria” variety sugars and acid content did influence the appreciation, for “Autumn Royal” the appreciation was not strongly correlated to wavelengths linked to sugar and acids; therefore, for this latter variety, the appreciation mainly relies on other parameters. The results of this work show how it is possible to extract valuable information from NIR spectra and search for a link between chemical composition in terms of sugar and acidic content and quality parameters even from intact berries using adequate chemometric techniques. This paper provides a good basis for more elaborate studies to correlate sensory properties and chemical composition of food.

## Figures and Tables

**Table 1:** Quality of the models obtained with the PLS algorithm. TSS: total soluble solid content; TA: titratable acidity; RMSECV: root mean square error of cross validation.

Parameter	RMSECV	Rank	Regions (cm^−1^)	Preprocessing	R^2^ (%)	Bias
**TA**	0.861	5	9400–6100 5452–4600	Vector normalization	57.32	−0.000762
**TSS**	1.3	6	9400–7500	First derivative + Vector normalization	83.04	0.01

**Table 2:** Maturity parameters and consumers’ acceptance.

	Maturity Parameters ^†^			Sensory Data ^‡^	
Variety	° Brix	TA	TSS/TA	%Acceptance	Preference
**Autumn royal**	18.3 ± 0.1 ^a^	3.8 ± 0.1 ^e^	49	100	7.13 ± 0.63 ^a^
	16.4 ± 0.1 ^b^	4.5 ± 0.1 ^d^	36	81	6.42 ± 0.58 ^ab^
	14.4 ± 0.0 ^c^	5.5 ± 0.1 ^ab^	26	50	5.63 ± 0.85 ^ab^
	13.8 ± 0.1 ^d^	5.0 ± 0.1 ^c^	27	56	5.17 ± 0.68 ^ab^
	13.3 ± 0.1 ^e^	5.0 ± 0.1 ^c^	27	33	5.25 ± 1.41 ^ab^
	12.6 ± 0.2 ^f^	5.7 ± 0.1 ^a^	22	29	4.40 ± 1.29 ^b^
	11.8 ± 0.2 ^g^	5.4 ± 0.1 ^b^	22	44	4.56 ± 1.88 ^b^
	**° Brix**	**TA**	**TSS/TA**	**%Acceptance**	**Preference**
**Victoria**	16.9 ± 0.1 ^a^	3.8 ± 0.1 ^d^	45	86	6.72 ± 1.85 ^a^
	14.9 ± 0.1 ^b^	3.9 ± 0.1 ^d^	38	86	6.08 ± 1.23 ^a^
	14.2 ± 0.1 ^b^	5.4 ± 0.1 ^c^	26	87	5.80 ± 1.50 ^a^
	11.9 ± 0.1 ^c^	5.3 ± 0.0 ^c^	22	40	4.20 ± 0.60 ^b^
	11.6 ± 0.1 ^c^	3.0 ± 0.0 ^e^	39	65	4.70 ± 0.50 ^b^
	10.6 ± 0.0 ^d^	6.7 ± 0.2 ^b^	16	32	4.08 ± 1.81 ^b^
	9.0 ± 0.1 ^e^	10.7 ± 0.1 ^a^	8	4	2.41 ± 1.29 ^b^

Values are mean ±SD. For each variety values in the same column bearing different letters are significantly different. Data were analyzed with ^†^ an ANOVA test followed by a Tukey post hoc test or ^‡^ the Kruskal–Wallis non-parametric test followed by the Dunn post hoc test using a *p*-value < 0.05.
